# Supplementary material for: Diverse evolutionary pathways challenge the use of collateral sensitivity as a strategy to suppress resistance
Source: eLife. 2023 Sep 22;12:e85023. doi: 10.7554/eLife.85023 (PMC10695565; doi:10.7554/eLife.85023)
Supplement: Supplementary file 1. [file elife-85023-supp1.docx]

**Supplementary File 1. Supplementary tables with additional experimental information for ‘Diverse evolutionary pathways pose a challenge to the use of collateral sensitivity as a strategy to suppress resistance.’**

**Supplementary file 1a:** **DHODH mutant lines tested against Tres Cantos Antimalarial Set compounds**

| **DHODH Mutation(s)** | **Parental Line** | **Reference** |
| --- | --- | --- |
| **E182D*** | 3D7 NIH | Ross et al., 2014 |
| **F227I*** | Dd2 | Ross et al., 2014 |
| **F227I/L527I*** | Dd2 | Ross et al., 2014 |
| **F227L** | 3D7 A10 | Mandt et al., 2019 |
| **F227L/L531F** | 3D7 A10 | Mandt et al., 2019 |
| **F227Y** | 3D7^0087/N9^ | Mandt et al., 2019 |
| **I263F*** | Dd2 | Ross et al., 2014 |
| **C276Y** | 3D7 A10 | Mandt et al., 2019 |
| **L531F*** | Dd2 | Ross et al., 2014 |

*****Dose response of indicated lines were previously reported in Ross et al., 2018 [45]

**Supplementary file 1b: DHODH genotype and corresponding dose response phenotype for all *in vitro* TCMDC-125334 selected lines**

| **Clone ID** | **DHODH Mutation(s)^1^** | **DHODH CNV** | **TCMDC-125334 EC_50_ (nM)** | **DSM265**  **EC_50_ (nM)** | **IDI-6273 EC_50_ (nM)** | **Genz669178 EC_50_ (nM)** |
| --- | --- | --- | --- | --- | --- | --- |
| 3D7 A10 | WT | 1 | 140±42.6 | 4.5±1.6 | 502±209 | 6.9±2.2 |
| T-F1-C1 | WT | 2 | 280±81.3 | 8.2±2.6 | 993±220 | 16.9±6.70 |
| T-F1-C2 | WT | 3 | 426±146** | 12.5±2.36* | 1597±212* | 27.0±10.0* |
| T-F1-C3 | WT | 2 | 298±36.5 | 8.7±0.37 | 1330±234 | 17.6±7.90 |
| T-F2-C1 | I263S | 1 | 432±130.8** | 2.5±0.74 | 7057±671** | 26.7±15.5* |
| T-F2-C2 | WT | 1 | 137±25.1 | 4.3±0.94 | 572±91.5 | 7.79±3.42 |
| T-F2-C3 | WT | 1 | 202±60.9 | 4.1±1.7 | 515±103 | 7.91±3.24 |
| T-F2-C4 | WT | 1 | 161±28.6 | 5.1±1.2 | 692±211.1 | 8.03±0.729 |
| T-F2-C5 | WT | 1 | 157±33.9 | 3.0±0.70 | 404±51.3 | 6.10±3.00 |
| Overall P-value (approximate) | | | 0.0004 | 0.0013 | 0.0007 | 0.0013 |
| Kruskall-Wallis Statistic | | | 28.52 | 25.54 | 27.05 | 25.50 |

The parasite lines are each designated with a unique identifier; for example, T-F1-C1 was selected with TCDC-125334 (T) came from Flask 1 (F1), and is designated as “C1” for “Clone 1”. Data is shown as mean EC_50_ +/- standard deviation. Statistical significance was determined by a Kruskal-Wallis test, with post-hoc multiple comparisons (Dunn’s) of each clone to 3D7 A10. *P ≤ 0.05; **P ≤ 0.01. Overall statistics are reported for each comparison group. Each dose response assay was performed with triplicate technical replicates, and average EC_50_’s were obtained from 3-4 independent biological replicates.

^1^Variants identified by whole-genome sequencing

**Supplementary file 1c: Percentage of whole-genome sequencing reads calling a DHODH mutant allele in bulk populations selected with DSM265**

|  | **C276Y** | **I273M** |
| --- | --- | --- |
| Flask 1 | 5% | 14% |
| Flask 2 | 97% | 0.3% |
| Flask 3 | 0% | 44.9% |

| **Clone ID** | **DHODH Mutation(s)^1^** | **DHODH CNV** | **TCMDC-125334 EC_50_ (nM)** | **DSM265**  **EC_50_ (nM)** | **IDI-6273 EC_50_ (nM)** | **Genz669178 EC_50_ (nM)** |  |
| --- | --- | --- | --- | --- | --- | --- | --- |
| 3D7 A10 | WT | 1 | 140±42.6 | 4.5±1.6 | 502±209 | 6.9±2.2 |  |
| C276Y Parent | C276Y | 1 | 12.40±3.089**** | 63.9±19.84 | 41.7±12.7**** | 22.0±6.68 |  |
| D-T-F1-C1 | C276Y | 3-4 | 50.3±10.4*** | 439.3±115.96* | 185±36.1 | >106 |  |
| D-T-F1-C2 | C276Y | 4 | 73.0±14.7 | 611±159* | 345±64.1 | >106 |  |
| D-T-F1-C3 | C276Y | 4 | 45.9±11.2* | ND | 199±58.6 | >129 |  |
| D-T-F1-C4 | C276Y | 2-3 | 54.5±21.0 | ND | 191±95.4 | >230 |  |
| D-T-F2-C1 | C276Y | 2 | 31.1±6.89**** | 196±56.6 | 138±44.0* | 117±30.0 |  |
| D-T-F2-C3 | C276Y | 2-3 | 43.0±7.81* | ND | 164±12.0 | >106 |  |
| D-T-F2-C4 | C276Y | 4 | 60.2±22.0 | ND | 232.0±39.2 | ND |  |
| D-T-F2-C2 | C276Y | 4 | 77.2±25.1 | 801±270 | 326±155 | ND |  |
| D-T-F3-C1 | C276Y/F227Y | 1 | 65.6±22.9* | 9042±2985**** | 163±36.3** | 69.7±14.5 |  |
| D-T-F3-C2 | C276Y/F227Y | 1 | 76.3±27.7 | 9960±3923**** | 183±84.3 | 97±54.5 |  |
| D-T-F3-C3 | C276Y/F227Y | 1 | 53.6±10.0* | >499 | 174±23.74 | 90.5±34.4 |  |
| Overall P-value (approximate) | | | <0.0001 | <0.0001 | <0.0001 | <0.0001 |  |
| Kruskall-Wallis Statistic | | | 38.12 | 47.36 | 45.55 | 54.32 |  |

**Supplementary file 1d: DHODH genotype and corresponding dose response phenotype for all *in vitro* selection of DHODH C276Y parent with TCMDC-125334**

The parasite lines are each designated with a unique identifier; for example, D-T-F1-C1 was selected first with DSM265 (D), then from TCMDC-125334 (T), came from Flask 1 (F1), and is designated as “C1” for “Clone 1”. Data is shown as mean EC_50_ +/- standard deviation. Statistical significance was determined by a Kruskal-Wallis test, with post-hoc multiple comparisons (Dunn’s) of each clone to 3D7 A10. *P ≤ 0.05; **P ≤ 0.01; ***P≤ 0.001; ****P≤ 0.0001. Overall statistics are reported for each comparison group. Each dose response assay was performed with triplicate technical replicates, and average EC_50_’s were obtained from 3-8 independent biological replicates.

WT=Wildtype

^1^Variants identified by whole-genome sequencing

ND indicates that the EC_50_ could not be determined; parasites were resistant to the range of doses tested as indicated by lack of complete kill at the highest dose of 500nM. A representative set of clones were tested at higher concentrations

**Supplementary file 1e: DHODH genotype and corresponding dose response phenotype for all *in vitro* TCMDC-125334 + DSM265 selected lines**

| **Clone ID** | **DHODH Mutation(s)** | **DHODH CNV** | **TCMDC-125334 EC_50_ (nM)** | **DSM265**  **EC_50_ (nM)** | **IDI-6273 EC_50_ (nM)** | **Genz669178 EC_50_ (nM)** |
| --- | --- | --- | --- | --- | --- | --- |
| 3D7 A10 | WT | 1 | 140±42.6 | 4.5±1.6 | 502±209 | 6.9±2.2 |
| DT-F1-C1 | WT^1^ | 2 | 260±79.5 | 8.6±1.6 | 1190±276 | 15.9±3.61* |
| DT-F1-C2 | WT^2^ | 1 | 153±21.0 | 6.1±0.88 | 628±117 | 7.74±1.23 |
| DT-F1-C3 | WT^1^ | 2 | 191±64.0 | 7.5±0.07 | 895±349 | 11.0±3.74 |
| DT-F1-C4 | WT^2^ | 2 | 319±137 | 11.2±4.5 | 1650±655* | 19.5±8.39* |
| DT-F2-C1 | V532A^1^ | 1 | 661±145** | 79.2±19.0* | 1320±368* | 11.4±3.08 |
| DT-F2-C2 | V532A^1^ | 1 | 536±54.4 | 82.4±23.1* | 881±185 | 8.69±2.35 |
| DT-F2-C3 | V532A^2^ | 1 | 816±480* | 93.2±36.9** | 1380±669* | 12.8±6.32 |
| DT-F2-C4 | V532A^2^ | 1 | 676±164** | 94.2±17.2** | 1330±410* | 12.0±3.67 |
| DT-F2-C5 | V532A^2^ | 1 | 556±142* | 73.4±16.4* | 1030±347 | 9.13±1.45 |
| DT-F2-C6 | V532A^2^ | 1 | 674±118** | 87.6±25.3** | 1310±412* | 14.4±4.16 |
| Overall P-value (approximate) | | | <0.0001 | <0.0001 | 0.0019 | 0.0091 |
| Kruskall-Wallis Statistic | | | 35.72 | 35.80 | 27.85 | 23.50 |

The parasite lines are each designated with a unique identifier; for example, DT-F1-C1 was selected with DSM255 and TCDC-125334 (DT) came from Flask 1 (F1), and is designated as “C1” for “Clone 1”. Data is shown as mean EC_50_ +/- standard deviation. Statistical significance was determined by a Kruskal-Wallis test, with post-hoc multiple comparisons (Dunn’s) of each clone to 3D7 A10. *P ≤ 0.05; **P ≤ 0.01. Overall statistics are reported for each comparison group. Each dose response assay was performed with triplicate technical replicates, and average EC_50_’s were obtained from 3 independent biological replicates.

WT= Wildtype

^1^DHODH genotype determined by whole-genome sequencing

^2^DHODH genotype determined by Sanger sequencing

**Supplementary file 1f: Whole-genome sequencing reads of DHODH V532A allele during competitive growth experiment**

|  |  | Wildtype Reads | V532A Reads | % V532A |
| --- | --- | --- | --- | --- |
| **Flask 1** | Day 0 | 136 | 112 | 45% |
|  | Day 8 | 189 | 206 | 52% |
|  | Day 17 | 164 | 198 | 55% |
|  | Day 28 | 233 | 196 | 46% |
| **Flask 2** | Day 0 | N/A | N/A | N/A |
|  | Day 8 | 193 | 204 | 51% |
|  | Day 17 | 227 | 258 | 53% |
|  | Day 28 | 204 | 173 | 46% |
| **Flask 3** | Day 0 | N/A | N/A | N/A |
|  | Day 8 | 191 | 217 | 53% |
|  | Day 17 | 229 | 269 | 54% |
|  | Day 28 | 249 | 216 | 46% |

**Supplementary file 1g: Whole-genome sequencing statistics**

| **Sample Name** | **Aligned Reads** | **Mean Whole Genome Coverage** | **Percent Bases Covered by 5 or More Reads** |
| --- | --- | --- | --- |
| 3D7-A10-Parent | 55929898 | 170.44 | 98.8 |
| 3D7-DSM265-F1 | 54755311 | 176.06 | 98.8 |
| 3D7-DSM265-F2 | 49779616 | 158.96 | 98.6 |
| 3D7-DSM265-F3 | 46611863 | 146.6 | 98.5 |
| TCMDC125334-3D7-T-F1-C1 | 13462259 | 43.45 | 93 |
| TCMDC125334-3D7-T-F1-C2 | 39262686 | 128.49 | 98.7 |
| TCMDC125334-3D7-T-F1-C3 | 37926711 | 127.01 | 98.5 |
| TCMDC125334-3D7-T-F2-C1 | 41671469 | 134.97 | 98.5 |
| TCMDC125334-3D7-T-F2-C2 | 46419210 | 153.23 | 98.7 |
| TCMDC125334-3D7-T-F2-C3 | 49679017 | 156.84 | 98.8 |
| TCMDC125334-3D7-T-F2-C5 | 71155194 | 205.79 | 98.7 |
| TCMDC125334-3D7-T-F2-C4 | 44909308 | 145.88 | 98.6 |
| DHODH-C276Y-Parent | 68749240 | 199.72 | 98.9 |
| TCMDC125334-C276Y-T-F1-C1 | 54058057 | 168.14 | 98.6 |
| TCMDC125334-C276Y-T-F1-C3 | 49952158 | 161.41 | 98.6 |
| TCMDC125334-C276Y-T-F1-C2 | 58034702 | 182.84 | 98.7 |
| TCMDC125334-C276Y-T-F1-C4 | 63741384 | 198.26 | 98.7 |
| TCMDC125334-C276Y-T-F2-C1 | 59905480 | 189.43 | 98.7 |
| TCMDC125334-C276Y-T-F2-C3 | 50442129 | 165.79 | 98.5 |
| TCMDC125334-C276Y-T-F2-C2 | 66531226 | 205.51 | 98.8 |
| TCMDC125334-C276Y-T-F2-C4 | 71408182 | 211.07 | 98.8 |
| TCMDC125334-C276Y-T-F3-C1 | 44669436 | 148.45 | 97.8 |
| TCMDC125334-C276Y-T-F3-C2 | 77516579 | 236.97 | 98.8 |
| TCMDC125334-C276Y-T-F3-C3 | 65068538 | 206.84 | 98.7 |
| 3D7-1µM-TCMDC125334-Pulse2-F1 | 45344143 | 119.96 | 98.6 |
| 3D7-1µM-TCMDC125334-Pulse2-F3 | 58687789 | 179.22 | 98.6 |
| TCMDC125334-DSM265-3D7-DT-F1-C1 | 55428568 | 149.81 | 98.1 |
| TCMDC125334-DSM265-3D7-DT-F1-C3 | 48467511 | 134.18 | 98.6 |
| TCMDC125334-DSM265-3D7-DT-F2-C1 | 44220447 | 115.61 | 98.4 |
| TCMDC125334-DSM265-3D7-DT-F2-C2 | 54256204 | 137.56 | 98.5 |
